# A database for retrieving information on SARS-CoV-2 S protein mutations based on correlation network analysis

**DOI:** 10.1186/s12863-022-01052-y

**Published:** 2022-05-04

**Authors:** Yoshiyuki Ogata, Ruri Kitayama

**Affiliations:** Graduate School of Agriculture, Osaka Metropolitan University, Sakai, Osaka 599-8531 Japan

**Keywords:** Database, SARS-CoV-2, Mutation, Correlation network, Mu variant

## Abstract

**Background:**

Over a million genomes and mutational analyses of SARS-CoV-2 are available in public databases, which reveal the phylogenetic tree of the virus. Although these data have enabled scientists to closely track the evolution and transmission dynamics of the virus at global and local scales, the Mu variant, recently identified in infections in South America, shows an unusual combination of mutations, and it is difficult to visualize these atypical characteristics in public databases based on a phylogenetic tree.

**Results:**

The Vcorn SARS-CoV-2 database was constructed to provide information on COVID-19 infections and mutations in the S protein of the virus based on correlation network analysis. A correlation network was constructed using the recall index of one mutation to another mutation. The network includes several network modules in which nodes represent mutations and are tightly connected to each other. Individual network modules contain mutations of single variants, such as the alpha and delta variants. In the network constructed to emphasize mutations of the Mu variant using the database, the mutations were found to be located in multiple network modules, indicating that the mutations of the variant may have originated from multiple variants or be located at a basal position with a high frequency of mutation.

**Conclusions:**

Vcorn SARS-CoV-2 provides information on COVID-19 and S protein mutations of SARS-CoV-2 via correlation network analysis. The network based on the analysis illustrates the unusual S protein mutations of the Mu variant. The database is freely available at http://www.plant.osakafu-u.ac.jp/~kagiana/vcorn/sarscov2/.

## Background

The SARS-CoV-2 virus has been causing a pandemic since the beginning of 2020. Many reports on COVID-19 and genomic analyses of the virus have been published [1–3]. Over a million genomes and mutational analyses of the virus are provided in public databases such as the NCBI GenBank virus database [4] and Nextstrain [5], and tens of thousands of new genomes are being uploaded daily [6]. These data have enabled scientists to closely track the evolution and transmission dynamics of the virus at global and local scales [7].

There are several public databases that contain information on the mutations of the virus, such as GenBank, Nextstrain, and SARS-CoV-2 MAT [8]. These databases provide a phylogenetic tree of the virus at the scale of the whole lineage or the local lineages based on analyses of viral mutations and the metadata of viral specimens. GenBank and Nextstrain associate variants of the virus with the geographical locations from which specimens infected by the variants were collected. SARS-CoV-2 MAT provides a daily updated environment by efficiently processing and formatting viral datasets.

Recently, the Mu variant (B.1.621 in the Pango lineage) [9] was designated as a variant of interest (VOIs) by the World Health Organization (WHO). Although the variant is not globally distributed to as great an extent as variants of concern (VOCs), such as the alpha (B.1.1.7) and delta (B.1.617.2) variants, it shows atypical mutation types in its S protein [10]. The alpha and delta variants have generally shown rampant spreading in different areas (i.e., England and India, respectively), and they thus exhibit different types of mutations in their S proteins. The Mu variant harbors some mutations similar to those of both VOCs according to the mutation list of the outbreak.info database [11]. The visualization of such unusual changes is useful for understanding the evolutionary traits of mutation in the virus. However, it is difficult to illustrate the separation of the mutations among multiple variants using a phylogenetic lineage.

Network graphs have been used to visualize complex systems [12–15]. Using this technique, Sekizuka et al. [16] depicted phylogenetic networks of SARS-CoV-2. The technique was also applied for correlation network analysis to detect local communities or network modules, in which nodes are tightly connected to each other. Correlation network analyses have been used to illustrate biological networks such as gene coexpression [17, 18] and gene homology networks [19–21]. To depict the relationships between the mutations shared by sampled virus genomes, a correlation network approach is useful.

Therefore, we visualized the different mutation types in the S protein of the virus via correlation network analysis by focusing on the ratio of specimens that share mutations. In the network, the mutations found in the alpha and delta variants formed network modules in which nodes represented mutations and were tightly connected to each other. However, the mutations of the Mu variant were distributed in multiple network modules. The obtained correlation network of S protein mutations allows us to discuss the evolutionary traits of a variant with mutations originating from multiple variants, such as the Mu variant.

## Initial steps

The Utility and discussion section provide the detailed steps for retrieving information from the Vcorn SARS-CoV-2 database. In this section, the steps for quick retrieval are explained.

1. Global cases: 1) select a week (e.g., Week 113), 2) select a map or all maps (e.g., All maps), and then 3) click the “submit” button.
2. Domestic cases: 1) select a nation (country/territory/area/district/province; e.g., United States), 2) select a period (daily/weekly; e.g., daily), and then 3) click the “submit” button.
3. Search for WHO labels: 1) select a WHO label (e.g., Mu) and then 2) click the “submit” button.
4. Search for single S protein mutation: 1) select or input a mutation code (e.g., D614G), and then 2) click the “submit” button.
5. Search for multiple S protein mutations: 1) select a mutation code (e.g., N501Y), 2) select another mutation code (e.g., T95I), and then 3) click the “submit” button.

## Development

**Fig. 1:**
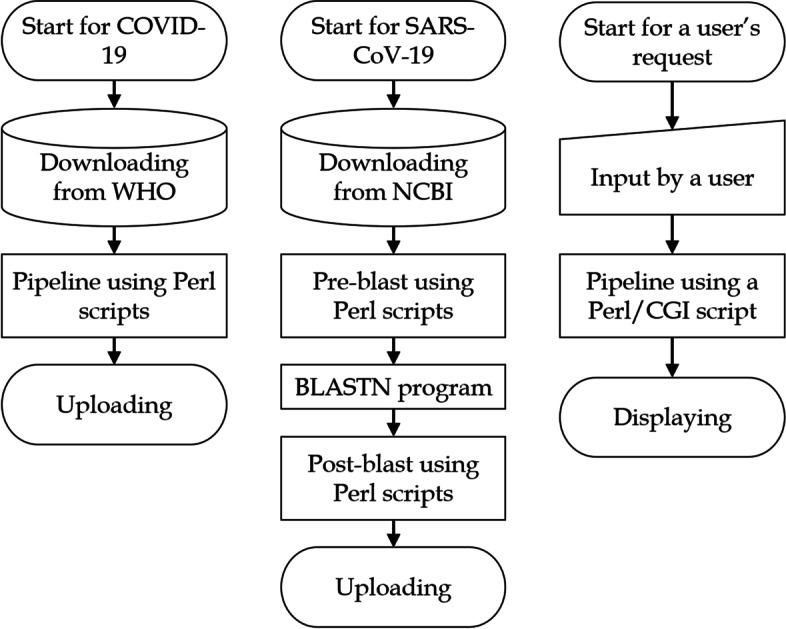
Flowchart providing an overview of the system

In this section, an overview of the Vcorn SARS-CoV-2 database system is provided (Fig. 1). The sources of most of these data were the WHO Coronavirus Dashboard (https://covid19.who.int/info/), for information on COVID-19 cases and deaths, and the NCBI Virus SARS-CoV-2 Data Hub database (https://www.ncbi.nlm.nih.gov/sars-cov-2/), for information on SARS-CoV-2 genome data.

A pipeline including Perl scripts developed by the authors was used for processing the data on COVID-19. A pipeline combining the BLAST program of NCBI and the Perl scripts developed by the authors was used for processing the data on SARS-CoV-2. These pipelines have been published in the database and will be updated monthly (http://www.plant.osakafu-u.ac.jp/~kagiana/vcorn/sarscov2/21/pipeline.html).

A user’s input is processed by the Perl/CGI programs developed by the authors to access to a page of interest. These Perl programs were also published on the same website.

## Construction and content

### Pipeline

The pipeline for monthly updates has been published on the Vcorn SARS-CoV-2 website (http://www.plant.osakafu-u.ac.jp/~kagiana/vcorn/sarscov2/21/pipeline.html).

### Cases of and Deaths Caused by COVID-19

A COVID-19 dataset (https://covid19.who.int/WHO-COVID-19-global-data.csv) was downloaded from the WHO Coronavirus Dashboard every week. The numbers of cases of and deaths caused by COVID19 according to nation (or region/territory/district) were collected from the dataset. Although the WHO provided these numbers for Taiwan at the beginning of the outbreak of COVID-19, the numbers for Taiwan are included in those for China as of November 2021. However, Taiwan has implemented distinct precautions against the virus and shown specific tendencies of COVID-19, such as a peak from May to July 2021. Therefore, the present database contains the numbers of cases and deaths in Taiwan, which were obtained from the Taiwan Centers for Disease Control. Recently, the WHO Coronavirus Dashboard noted technical difficulties in datasets from the WHO European Region. Vcorn SARS-CoV-2 provides numbers based on the original datasets obtained from the dashboard.

Demographic datasets of COVID-19 cases by age group were obtained from the health organizations of France, Japan, South Korea, the United Kingdom, and the United States and were used to calculate weekly data by age group.

### Analyses of S protein mutations of SARS-CoV-2

Three types of viral genome datasets were obtained from the NCBI Virus SARS-CoV-2 Data Hub database (https://www.ncbi.nlm.nih.gov/sars-cov-2/): 1) base sequences of the genomes, 2) metadata of specimens including collection data and nations, and 3) a statistical dataset including each mutation. As of February 1st, 2022, 3,578,352 sequences (approximately the number of specimens) were available, and the main geographical locations from which these specimens were collected are shown in Table 1.

**Table 1:** Geographical locations of SARS-CoV-2 specimens and cases

Nation	Specimens (S)	Cases (C)	Ratio (S/C)
United States	1,816,065	73,958,288	2.4%
United Kingdom	1,307,360	17,405,439	7.5%
Germany	276,777	9,978,146	2.7%
Switzerland	99,615	2,268,533	4.3%
Australia	13,312	2,182,390	0.6%
Slovakia	10,409	1,022,784	1.0%
Iceland	5,365	67,594	7.9%
Bahrain	4,946	374,575	1.3%
Kenya	4,013	321,381	1.2%
Mexico	2,950	5,198,891	< 0.1%
Denmark	2,599	1,742,569	0.1%
India	2,552	41,469,499	< 0.1%
New Zealand	2,427	16,246	14.9%
Japan	2,195	2,730,828	< 0.1%
Hong Kong	1,853	–	–
France	1,712	18,634,317	< 0.1%
Estonia	1,681	344,060	0.4%
Netherlands	1,562	4,420,834	< 0.1%
Puerto Rico	1350	457,501	0.2%
Bangladesh	1,233	1,811,987	< 0.1%
Poland	1,219	4,925,158	< 0.1%
Spain	1,184	10,100,109	< 0.1%
Brazil	1,121	25,348,797	< 0.1%
Thailand	1,106	2,447,964	< 0.1%
Saudi Arabia	1,090	687,264	0.1%
Egypt	1,072	425,911	0.2%
Italy	1,063	10,983,116	< 0.1%

Nations providing one thousand or more specimens are listed. The numbers of specimens and cases were obtained from different data sources (NCBI Virus SARS-CoV-2 Data Hub and WHO Coronavirus Dashboard databases, respectively). Therefore, these specimens are not necessarily included in the number of cases

BLASTN analysis was performed by using the sequences as the query and the base sequences encoding the S protein as the database (reference sequences). The BTOP column was used to determine the mutation types of each sequence (i.e., the basic positions, base changes, deletions, and insertions of each mutation). These base changes were transformed into substitutions, deletions, or insertions of amino acids.

The earliest specimen that contained each mutation was determined based on the metadata of the specimens. Since it was possible for the collection date of a specimen to be unreasonable (e.g., a date before the outbreak), an outlier test was performed on the collection dates of the mutations. This test can provide another type of earliest specimen for each mutation that may be more reasonable than the original type.

The mutations were classified into two categories, i.e., major, and minor mutations. When the number of specimens that contained a mutation represented 1% or more of all specimens studied, the mutation was classified as a major mutation, while when the number of specimens with a certain mutation accounted for less than 1%, the mutation was classified as a minor mutation.

A list of specimens that contained a major mutation of interest (referred to as “Mutation A”) was compared with a list of specimens that contained another major mutation (“Mutation B”), and the recall index of Mutation A to Mutation B (${R}_{AB}$) was calculated as follows:

$${R}_{AB}=\frac{{N}_{S}}{{N}_{A}}$$

where ${N}_{S}$ and ${N}_{A}$ represent the number of specimens sharing both Mutations A and B and the number of specimens containing only Mutation A, respectively. When the index was one, the numbers of specimens containing the two mutations were perfectly equivalent to each other, while when the index was 0.5, half of the specimens that contained Mutation A also contained Mutation B. A higher index value indicated that more specimens contained both mutations. The indices of all major mutations were calculated. A correlation network was constructed based on these indices using the Pajek tool [22]. In the network, a node represented a major mutation and was connected to another major mutation when the value is 0.5 or higher (an arrowhead represents Mutation A). When the correlation network was generated using 0.8 as the threshold of the recall index, the interconnections in the network were similar to those in the network obtained when the threshold was 0.5. In contrast, when the correlation network was generated using 0.1 as the threshold, the interconnections in the network were much more numerous than those in the network obtained when the threshold was 0.5. This indicates that although many major mutations tend to coexist in particular specimens, other specimens tend to contain none of these mutations. When both the index of Mutation A to Mutation B and its opposite index were 0.5 or higher, the direction based on a higher index was adopted to represent the possible process of evolution. For instance, when the former and latter indices were 0.6 and 0.7, respectively, the direction of the arrow was from Mutation A to Mutation B, indicating that Mutation B might occur after Mutation A based on the memberships of the specimens.

### Implementation

The scripts for data processing on our server were all written in Perl. The present database is available without user registration and can be viewed on common browsers and operation systems, such as Google Chrome, Microsoft Edge, FireFox, and Microsoft internet Explorer on Windows, Macintosh, iPhone, and Android systems. All illustrations are presented in SVG format. When a user accesses the web page on Internet Explorer, the illustrations are downloadable in SVG and PNG formats.

## Utility and discussion

### User interface

The portal website of Vcorn SARS-CoV-2 mainly provides three kinds of retrieval methods to acquire information on COVID-19 and SARS-CoV-2 S protein mutations: 1) global data on COVID-19, 2) domestic data on COVID-19, and 3) data on S protein mutations. With one or a few clicks, a user can access a web page that contains information of interest.

### Global data

In the “Global” section, there is a global heatmap in which each nation (or region, territory or district) can be clicked to access a web page with information on COVID-19 in that nation. The colors of the heatmap from yellow to black represent the cumulative number of COVID-19 cases in ascending order.

**Fig. 2:**
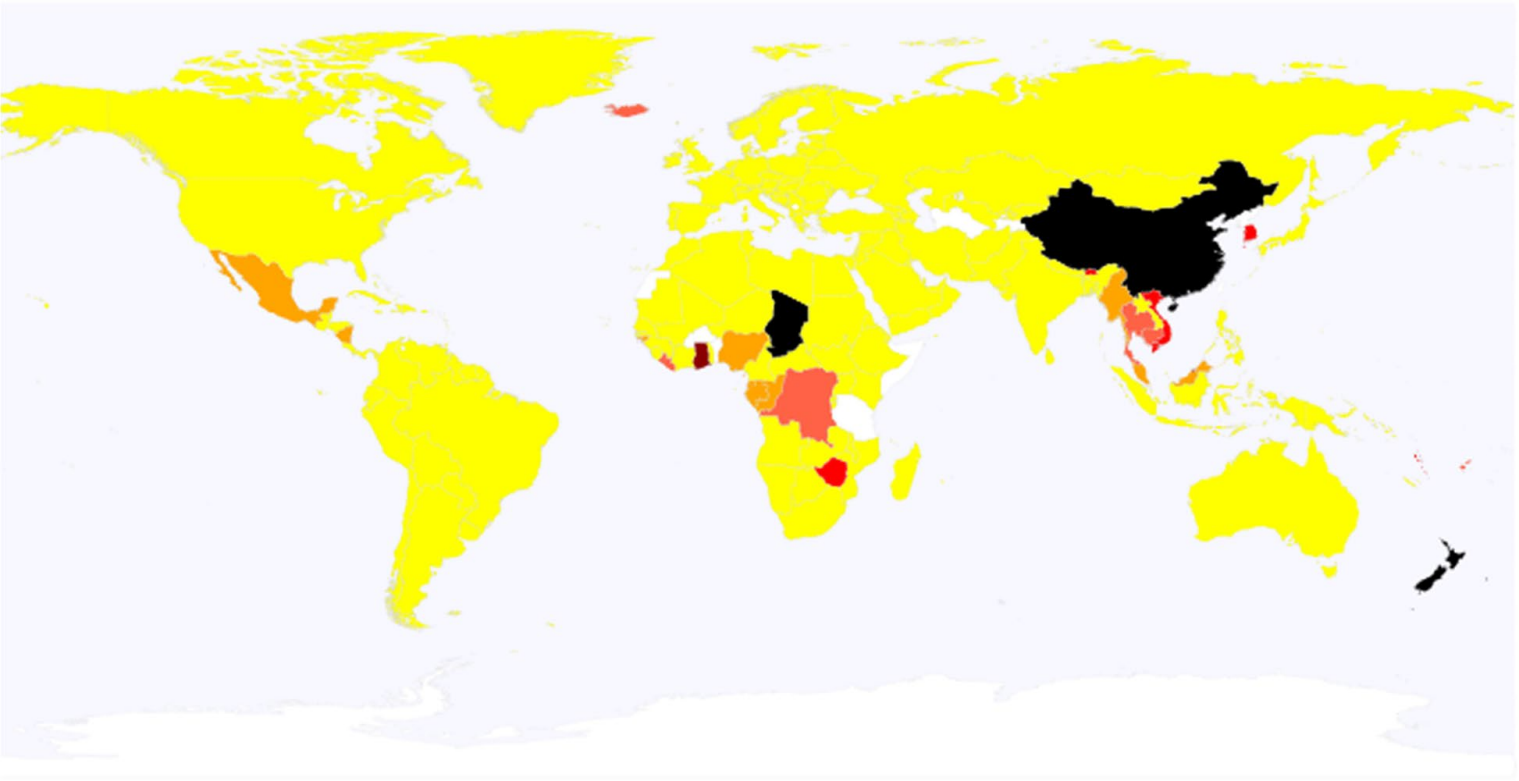
A global map showing the change in the number of COVID-19 cases. The change is based on the ratio of cases in the present week to those two weeks ago. In the map, the color for each nation depends upon the ratio; i.e., black, dark red, red, tomato, orange, and yellow represent 8 times or larger, 4 times or larger, twice or larger, 1.5 times or larger, equivalent or larger, lower than 1, respectively. Data for nations in white are unreported. This global map is based on a file obtained from Wikipedia (https://en.wikipedia.org/wiki/Wikipedia:Blank_maps), according to its Terms of Use

In the “Global cases” section, just three steps are required to access to a web page with six types of global maps, including maps of 1) weekly cases, 2) weekly deaths, 3) the change in cases (the ratio of cases in the present week to the cases two weeks ago) (Fig. 2), 4) cumulative cases, 5) cumulative cases per million persons, and 6) the monthly mortality ratio (the ratio of monthly deaths to monthly cases).

### Domestic data

In the “Domestic cases” section, just three steps are required to access two kinds of web pages with daily and weekly data on COVID-19 in the selected nation. On the web page for daily cases and deaths, line charts of daily cases and deaths are depicted in blue and red, respectively. The scale of the chart of cases (the left vertical axis) is ten times higher than that of deaths (the right vertical axis). The duration of the line charts is from the beginning of 2020 to the end of 2022. The data table includes not only the daily cases and deaths but also the S protein mutations that were assigned to collection dates according to the metadata of the specimens, with hyperlinks to web pages for the specific mutations.

**Fig. 3:**
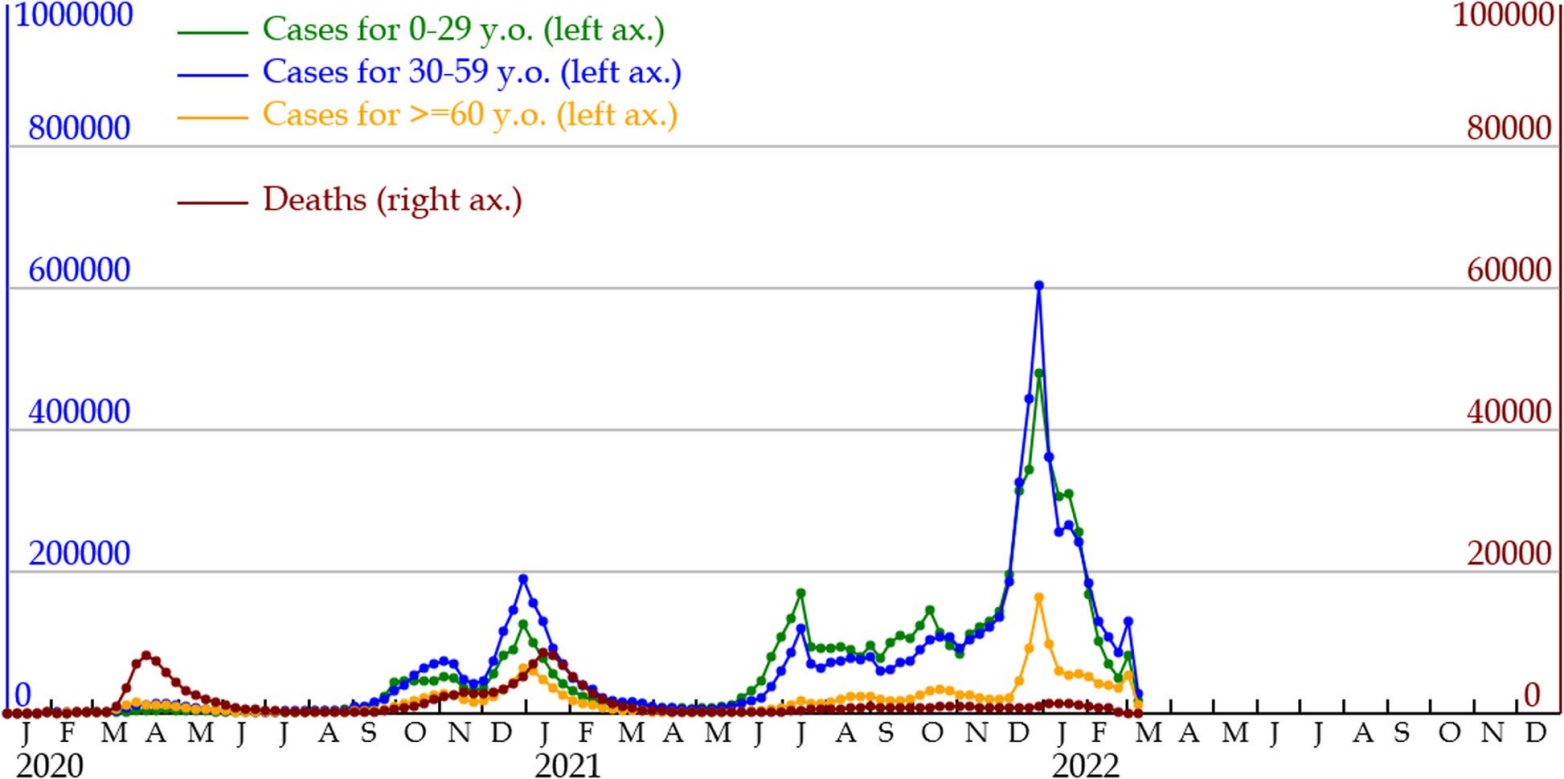
Line charts of COVID-19 cases by age group in England. The horizontal axis represents months from 2020 to 2022. Left and right vertical axes represent the numbers of cases and deaths, respectively. The former axis is ten times larger than the latter. Line charts in green, blue, and yellow represent monthly cases in the young (0–29 years old), middle (30–59 years old), and elderly (60 years old or older) age groups, respectively. The line chart in red represents monthly deaths

On the web page for weekly cases and deaths, the line charts are similar to those for the daily data. Cases divided by age group are depicted in line charts for France, Japan, South Korea, the United Kingdom, and the United States as of February 2022 (Fig. 3).

### Correlation network among major mutations

**Fig. 4:**
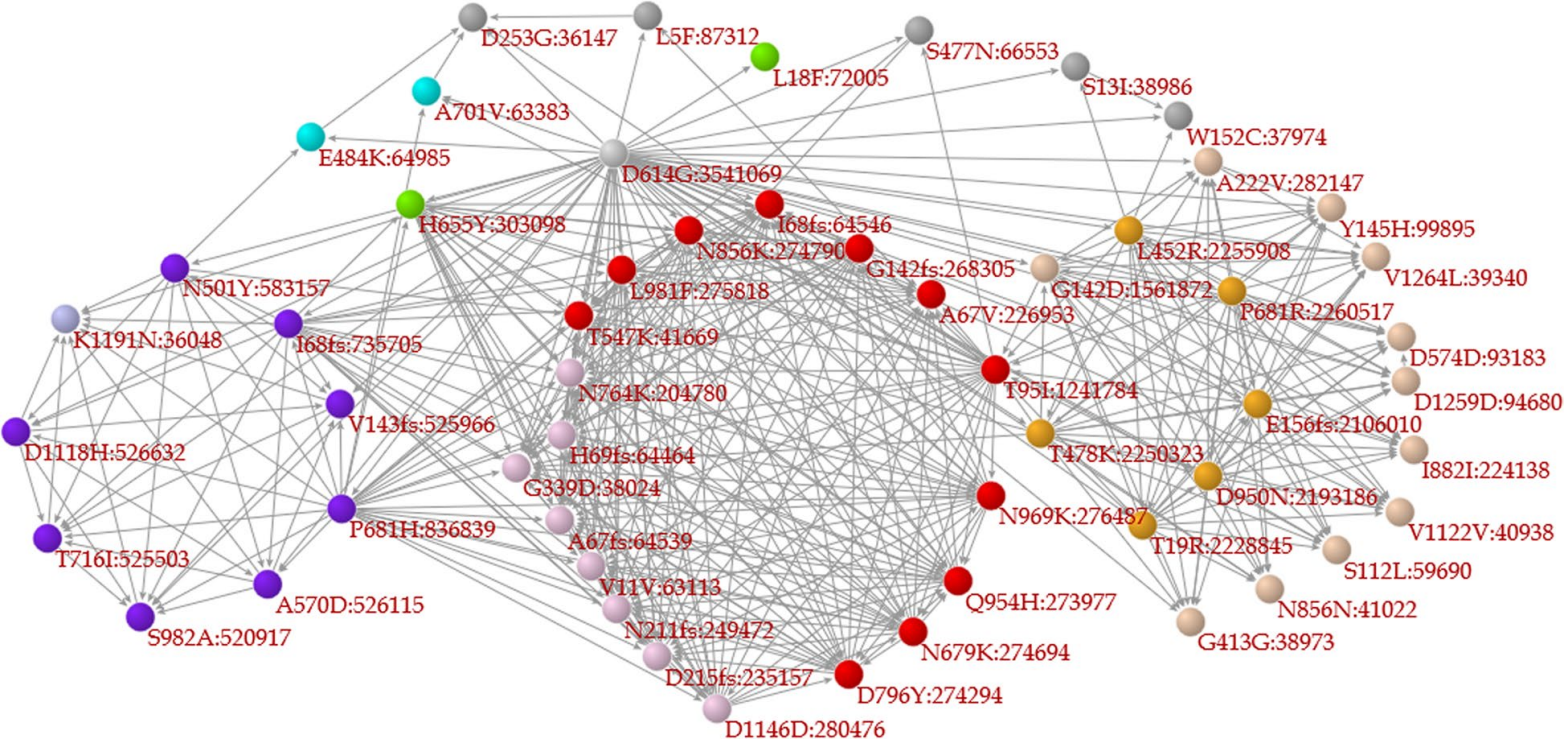
A correlation network composed of major mutations. When the frequency of a mutation is 1% or larger among the specimens studied, the mutation is classified as a major mutation in the present research. In the network, a node (Mutation A) represents a major mutation and is connected to another node (Mutation B) based on the recall index from Mutation A to Mutation B (${R}_{AB}\ge 0.5$). The coloration of the nodes depends upon the network modules, in which nodes are tightly connected to each other. Purple, orange, and red nodes represent mutations contained in Alpha, Delta, and Omicron variants, respectively. The direction of the arrow is based on the recall index from one node (Mutation B) to another node (Mutation A), in the opposite direction of the recall index

In the “Search for single S protein mutation” section, a user can click on a mutation in a correlation network of major mutations (Fig. 4) or select/input a mutation to access a web page for the mutation of interest. The web pages of S protein mutations contain 1) a list of variants with WHO labels containing the mutation of interest, 2) a correlation network among major mutations in which mutations related to the mutation of interest are labeled in red, 3) bar charts of the ratio of specimens sharing major mutations, 4) a monthly line chart of the temporal change in the global frequency of the mutation, and 5) the geographical pattern of specimens containing the mutation. In the correlation network and the bar graph illustrating mutation coexistence, each mutation is hyperlinked to the web pages relevant to that mutation.

In the “Search for multiple S protein mutations” section, a user selects a pair of mutations of interest to access a web page for that pair. Although the web page for the pair is identical to that for a single mutation, a correlation network composed of major mutations emphasizes mutations related to the pair; i.e., mutations that coexist (with a recall index of 0.5 or higher) in specimens that contain the mutation pair are labeled in red. When a user selects a pair of mutations of interest from different network modules, the specimens selected by this retrieval method may differ from VOCs such as alpha and delta variants but be similar to Mu variants.

### Variants with WHO labels

In the “Search for WHO label” section, a user selects a WHO label of a SARS-CoV-2 variant to access a web page for the variant, in which there is a correlation network composed of major mutations in which nodes represent major mutations and are connected to other mutations based on the recall index between mutations (0.5 or larger). The colors of the nodes depend on the network modules according to the Louvain method of the Pajek tool, and purple and blue nodes represent alpha and delta variants, respectively. Mutations with red or gray labels are present or not present in the variant, respectively.

### Case study

Two kinds of retrieval methods are exemplified in this section. One is a general strategy for obtaining information on domestic data on COVID-19, and the other is a method for searching for information on the Omicron variant.

In the Domestic cases section of the portal page, “United Kingdom” is selected in the first step, “Weekly” is selected in the second step, and the “Submit” button is clicked to access a web page for information on weekly COVID-19 data in the United Kingdom (only England) (Fig. 3). On the web page, line charts in green, blue, and yellow represent the weekly numbers of cases in 0 to 29 year olds (young age group), 30 to 59 year olds (middle age group), and 60 year olds and older (elderly age group), respectively, and a line chart in red represents the weekly numbers of deaths. According to the line charts, the United Kingdom has shown three peaks of COVID-19, from March to June 2020, September 2020 to March 2021, and May 2021 to the present. In the first peak, the number of deaths was quite high; e.g., in the week from April 8 to 14, 2020, the ratio of deaths to cases was 32.9%. In the second peak, the ratio was lower than that in the first peak, e.g., in the week from January 13 to 19, 2021, it was 3.4%. In the third peak, the ratio has been markedly lower than that in the second peak, e.g., in the week from September 8 to 14, 2021, it was 0.5%. It is possible that this low number of deaths may be due to intensive vaccination. The numbers of cases in the three age groups were different among these peaks. During the first peak, case numbers in the middle and elderly age groups were similarly high, and the number in the young age group was lower. After the first peak, case numbers in the young and middle age groups were larger than those in the elderly age group during the second and third peaks. Based on these line charts, it is simple to understand the tendency of cases by age group.

**Fig. 5:**
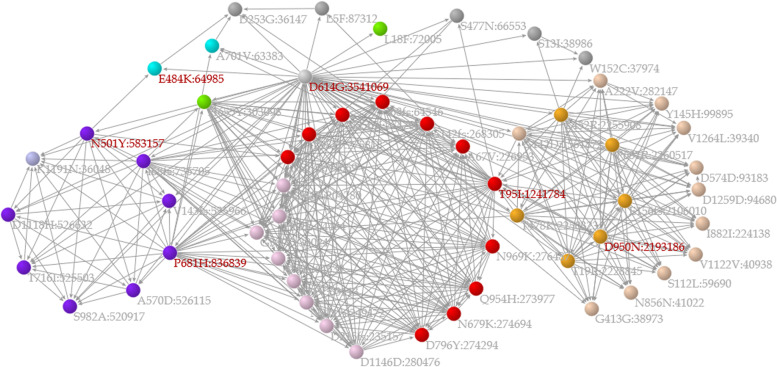
Correlation network emphasizing mutations contained in the Mu variant. The legends of the network are similar to those of Fig. 4, except for the coloration of the labels of mutations; i.e., a mutation with a red or gray label is present or not present in the variant, respectively. According to the network, the Mu variant has six major mutations, among which there are two nodes in purple (alpha variant), one node in orange (delta variant), and one node in blue

A way to retrieve information on Omicron variant is illustrated with an example in this paragraph. In the Search for WHO label section of the portal site, “Omicron” was selected in the first step, and the “Submit” button was then clicked. This mutation is included in all variants designated by the WHO that include the Mu variant. On the web page for D614G, a hyperlink to the Mu variant is clicked to show a web page for the variant (Fig. 5). The network shows that the mutations of the variant are separately located in different network modules; e.g., N501Y and P681H are located in a network module in purple (i.e., alpha variant), D950N is in an orange module (i.e., delta variant), and E484K is in a blue module. This indicates that the mutations of the Mu variant potentially originated from multiple variants. The unusual combination of the mutations contained in the variant is explicitly depicted in such a correlation network.

### Discussion

The Vcorn SARS-CoV-2 database provides information not only on daily and weekly COVID-19 data by nation/region/territory/district but also on mutations in the S protein of SARS-CoV-2 based on correlation network analysis. A correlation network that emphasizes mutations related to a mutation is constructed and helps us to understand the evolutionary traits of S protein mutations in a series of variants, particularly variants containing mutations found in multiple variants, as is the case for the Mu variant. In a correlation network that contains major mutations, several mutations (nodes) like D614G are the bases of many arrows; i.e., such mutations may precede other mutations. Mutations included in network modules, in which mutations are tightly connected to each other, are detected in sampled genomes similar to those harboring other mutations in the same modules, indicating that the timing of their occurrence was almost simultaneous to each other. Some variants with WHO labels, such as Alpha, Delta, and Omicron, tend to form network modules.

GenBank [4], Nextstrain [5], and SARS-CoV-2 MAT [8] also provide information on mutations of the virus and refined phylogenetic trees to help trace the lineage of the mutations and the variants. The outbreak.info database [11] provides well-organized and clear illustrations and charts to show the global and temporal situation of a variant. Forster et al. [23] emphasized that a network approach for drawing a phylogenetic tree was useful to trace routes of infection in COVID-19 cases during the first global outbreak and provided an example of a phylogenetic tree for tracing such cases and classifying three types of variants. Gupta et al. [24] applied a network approach to trace a lineage of variants and to understand gene functions in specimens of the virus. Sekizuka et al. [16] depicted a phylogenetic network of sampled virus genomes to identify potential infection routes. These databases and depictions are useful for discussing the relationships between sampled virus genomes. However, they have no explicit approach for addressing an unusual combination of mutations contained in a variant. Vcorn SARS-CoV-2 is suitable for visualizing such unusual combinations.

Although the mutations assigned to a given variant should be present in similar specimens, they tend to occur in different specimens to some extent. This may be caused by errors in sequence alignment during the BLASTN search and by biological and evolutionary events. The latter type of events may result from the combination of (or cross-talk between) mutations contained in multiple variants in a specimen and from differences in the frequency of mutation based on the S protein structure according to the positions of base sequences. When a base position shows a high frequency of mutation, the mutation at that position may occur in different cases. The Mu variant contains such mutations, as described in the previous paragraph [10]. However, its infectivity remains largely unknown [25]. The rational tracing of mutations contained in the variant is useful for understanding the evolutionary traits of possible mutations.

### Future developments

The Vcorn SARS-CoV-2 database is updated weekly with information on COVID-19 and monthly with information on mutations in the S protein of SARS-CoV-2. The Vcorn project aims to elucidate evolutionary traits in viruses, including not only SARS-CoV-2 but also other viruses, such as influenza virus, using correlation network approaches. In the near future, the project will perform correlation network analyses of all coronaviruses to shed light on the missing link between SARS-CoV-2 and its ancestral virus.

## Conclusion

Vcorn SARS-CoV-2 provides information on COVID-19 and S protein mutations of SARS-CoV-2 via correlation network analysis. The network based on the analysis allows the explicit visualization of the unusual combination of S protein mutations contained in the Mu variant. The database is freely available at http://www.plant.osakafu-u.ac.jp/~kagiana/vcorn/sarscov2/.

## Data Availability

Vcorn SARS-CoV-2 is a freely available database for all users with no login requirements and is available at http://www.plant.osakafu-u.ac.jp/~kagiana/vcorn/sarscov2/. The database is functional on all modern operating systems, such as Windows, Macintosh, iPhone, and Android, and web browsers, such as Microsoft Edge, Microsoft internet Explorer, Google Chrome, Mozilla Firefox, and Safari.

## Abbreviations

VOC: Variants of concern
VOI: Variants of interest
WHO: World Health Organization
